# Pancreatic cancer orthotopic graft in a murine model

**DOI:** 10.1590/acb382823

**Published:** 2023-08-04

**Authors:** Milena Muzzolini, Ismahane Belhabib, Victoire Cardot, Annemilaï Tijeras-Raballand, Cindy Neuzillet, Corinne Bousquet, Renato Micelli Lupinacci, Christine Jean

**Affiliations:** 1 Ambroise Paré Hospital – Oncologic and Metabolic Surgery – Department of Digestive – Boulogne-Billancourt, France.; 2 Paris Cité University Santé, France – Université des Sciences de la Santé – Santé, France.; 3 Université Toulouse III-Paul Sabatier – Université de Toulouse – Centre de Recherche en Cancérologie de Toulouse – Institut National de la Santé et de la Recherche Médicale – Toulouse, France.; 4 Institut Curie – Molecular Oncology Team – Paris, France.; 5 OncoMega – Paris, France.; 6 Versailles St-Quentin en-Yvelines/Paris Saclay University – UFR Simone Veil – Santé, France.; 7 Institut Curie Saint Cloud – Saint-Cloud, France.

**Keywords:** Transplants, Mice, Pancreatic Neoplasms, Methods

## Abstract

**Purpose:**

Pancreatic ductal adenocarcinoma (PDAC) is one of the deadliest cancers with increasing incidence. Even if progress have been made, the five-year overall survival remains lower than 10%. There is a desperate need in therapeutic improvements. In the last two decades, new *in-vitro* models have been developed and improved, including tridimensional-culture spheroids and organoids. However, animal studies remain mandatory in the upscaling before clinical studies. Orthotopic and syngeneic grafting is a robust model to test a drug efficiency in a tumor and its microenvironment.

**Methods:**

We described a method for orthotopic and syngeneic graft of KRAS mutated, p53 wildtype, 8305 cells in a C57BL/6J mouse model.

**Results:**

With this microsurgical method, 30 mice were grafted, 24 by a junior and six by a senior, resulting in 95,8 and 100% of (partial and total) successful tumoral implantation, respectively. Twenty mice underwent ultrasound follow-up. It was an efficient method for the tumoral growth evaluation. At day 16 after grafting, 85% of the tumors were detectable by ultrasound, and at day 22 all tumors were detected.

**Conclusions:**

The presented method appears to be a robust and reliable method for pre-clinical studies. A junior master student can provide positive results using this technique, which can be improved with training.

## Introduction

Incidence of pancreatic cancer is rising and is predicted to be the second cause of death by cancer after 2030^1^. Even if progresses have been made in the last decade, the five-year survival rate is inferior to 10%^2^. Less than 20% of the patients will receive a curative strategy by the association of surgery and cytotoxic chemotherapy^3^^-^ 5 with an expected three-year survival of 63%^3^^,^ 4. Metastatic patients, if fit, can benefit from either gemcitabine plus nab-paclitaxel or FOLFIRINOX with reported overall survival rate from 8,5 to 11,1 months respectively^6^^,^ 7. Thus, there is a desperate need in identifying new therapeutic strategies for pancreatic cancer.

The last three decades saw the development of models for drug screening and pre-clinical studies. Adding to historically two-dimensional (2D) culture of tumor cells, three-dimensional (3D) cultures, including co-cultures in spheroids and organoids, have been developed^8^^-^ 12. Despite the advantage that 3D culture can represent, only *in-vivo* models recapitulate the full spectrum of tumor development with the establishment of a tumor microenvironment comparable to the one of human tumors^13^^,^ 14. Indeed, pancreatic cancer is a complex tumor structure, and interactions between tumor cells and microenvironment promote tumor development and metastasis^15^^,^ 16. Orthotopic and syngeneic graft of tumor cells in an immunocompetent mouse is an interesting model to validate drug efficacy after *in-vitro* screening.

Here we aimed to describe an easy and reproducible method for orthotopic and syngeneic pancreatic cancer cell graft in mice. To illustrate the feasibility of this method, we analyzed the results of the first 24 orthotopic pancreatic cancer cell graft performed by our mastership student and six by an experienced team.

## Methods

All experiments were in accordance with institutional guidelines and European animal protection law and approved by the responsible government agency (agreement number 2019061900061441).

### Cells and reagents

Mouse pancreatic tumor cells are tumor cells isolated from p48-Cre LSL-Kras^G12D^ (p53 wild type) and generously provided by Dr. D. Saur (University Hospital Klinikum rechts an der Isar, Technische Universität München, Germany). Mouse tumor cells were maintained in Dubelcco’s Modified Eagle Medium (DMEM) from Sigma Aldrich (ref D6429) containing 10% foetal bovine serum (FBS) (Life Technologies) supplemented with L-Glutamine (L-glu, 2 mmol/L), penicillin and streptomycin (P/S). The day of the procedure, the correct number of cells (20,000 cells/mouse) was centrifuged at 1,200 rotation per minute (RPM) for 3 minutes, washed with phosphate buffer saline (PBS) and re-suspended in 20 uL of Dubelcco’s PBS from Sigma Aldrich (ref. 8537). Cells were kept on ice before injection for a maximum of 3 hours.

### Before procedure

Eight-week-old C57BL/6J female mice were dispatched after reception in cages up to five animals per cage. Room temperature was kept between 19 and 22 °C and humidity between 55 and 65%. Food and water were available *ad libitum* in each cage. Mice were monitored for a week in our facility, and, one day before the procedure, mice were shaved (left flank) with an electrical instrument.

### Procedure

Mice were treated (intra-peritoneal injection) with the analgesic buprenorphine (0,05 mg/kg prepared in PBS) at least 30 minutes before the procedure.

Mice were put under general anesthesia using isoflurane 2,5% by inhalation with a flow of 1 L/min. Maintain of the anesthesia was performed with a facial mask with a flow of 0,4 L/min. The whole procedure was performed on mice kept on a heating pad (37 °C). General anesthesia was considered adequate in the absence of any reaction/reflex after toe pinch. A droplet of an eye gel (Lubrithal) was added in both eyes of anesthetized mouse to avoid eye dehydration.

**Figure 1:**
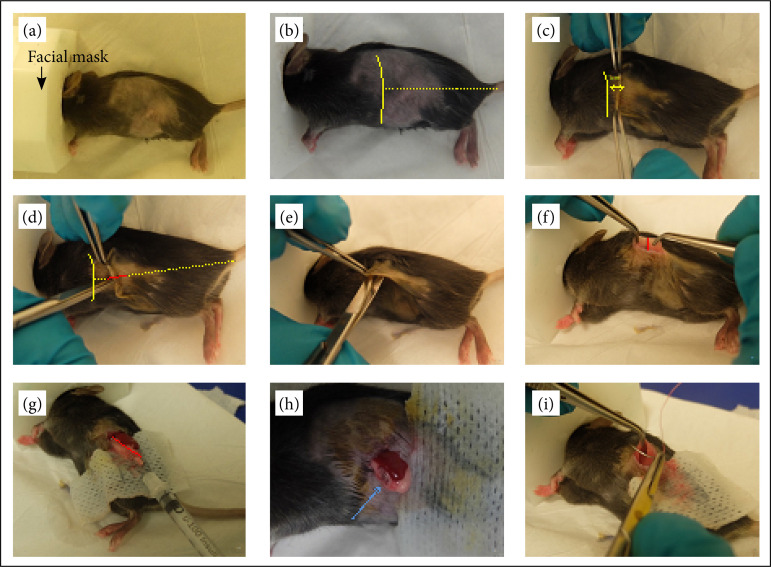
Orthotopic graft for pancreatic cells. **(a)** Mouse installation, black arrow shows the facial mask. **(b)** Pointer for a good incision are the grill cost and the beginning of the tail. **(c)** Incision 1 cm away from the grill cost. **(d)** Incision between two clinches. **(e)** Subcutaneous detachment. **(f)** Peritoneal incision. **(g)** Exposition and direction of the needle for injection. Red marker represents the splenic artery, above is the spleen and beneath is the pancreas. **(h)** Verification of the bubble formation (blue arrow). **(i)** Peritoneum closure. **(j)** Skin closure with a stapler.

Source: Authors.

Mice were placed on the right side, exposing the left flank to the operator. At this time, the correct positioning of the facial mask was verified (Fig. 1a). The skin was washed with ethanol 70% and then with iodide povidone.

A short incision (< 1 cm), 1 cm from the grill cost on a line between the grill cost and the start of the tail, was done using straight scissors. Incision was performed in the coronal plan in a head-to-toe direction between two clamps (Figs. 1b–1d). A subcutaneous detachment was performed in every four directions to facilitate the closure (Fig. 1e). The peritoneum, which is translucent in mice, was exposed. The skin could then be moved up- and downward easily, which facilitate the recognition of the spleen (dark red color) by transparency through the peritoneum. This maneuver allowed the incision of the peritoneum to be made just at the bottom of the spleen or just under (Fig. 1f). We used clamps to raise the peritoneum and avoid damage to the underlying organs. The spleen was extracted with a clamp ideally catching cautiously the bottom of the spleen to avoid bleeding. This gesture allows the concomitant exteriorization of the pancreas, which bounds to the spleen (Fig. 1g). The spleen and the pancreas were laid down over a sterile gauze to avoid spontaneous reintegration into the cavity and to allow correct recognition of the organs (the spleen is dark red, and one or more ectopic spleens might be present, the pancreas is beige, a bit whiter than the grease of the mesocolon and that the fatty tissue of the splenic hilum).

A total of 20,000 cells, kept on ice during the process and diluted in 20 μL of PBS, was injected in the pancreas using a needle (Micro-Fine U-100 insulin 0,5 mL) (Fig. 1g). The appearance of a bubble in the pancreas is the sign that the procedure was successful (Fig. 1h). After revision of the hemostasis, organs were re-integrated into the abdominal cavity by pushing the organs in the cavity and allowing them to regain their position by gravity.

The peritoneum was closed with absorbable 5/0 suture (Novosyn) (Fig. 1i). Skin was closed with a stapler and washed with iodide povidone. Animals were then removed from the facial mask and kept in warm conditions until complete awareness.

### After procedure

A second intra-peritoneal (IP) injection of buprenorphine (0,05 mg/kg) was performed 8 hours after the first one.

Mice behavior was verified in the evening of the procedure, in the next morning (in case of any sign of pain, a third injection of buprenorphine was performed IP) and then every day. Staples were removed at post operative day (POD) 8.

### Follow-up, tumor growth evaluation and mouse sacrifice

Mice were weighted twice a week. At POD 16, the presence of tumor within the pancreas was evaluated by ultrasound using Aixplorer (Supersonic) echograph and SL-22 transducer. The tumor volume follow-up was performed twice a week by ultrasound, and tumor volume was calculated using an ellipsoid model (volume = 4/3 **·** π* a/2 **·** b/2 **·** c/2; a, b and c being the diameters measured).

Total success was defined by the presence of a pancreatic tumor without subcutaneous tumor. Partial success was defined by the presence of a pancreatic tumor associated with a subcutaneous tumor. Failure was defined by the absence of any pancreatic tumor. End points were defined by either a weight loss greater than 20%, or a total tumor burden (subcutaneous tumor + pancreatic tumor) reaching more than 700 mm^3^ or the presence of any clinical worrisome feature (signs of suffering animal).

At the day of sacrifice, mice were put under general anesthesia, intracardiac punction was carried out to collect the mice blood, and a cervical dislocation was performed. The intracardiac punction was performed using a needle (#300600) inserted just above sternum (xiphoidal end) of the animal with an inclination of 45° in the direction of the head. The presence of blood within the needle (even before any aspiration) allows the identification of the right position within the cardiac cavity. The punction allows to collect up to 900*μL of blood.

At sacrifice, we systematically searched for any sign of peritoneal carcinomatosis or solid-organ metastasis. Liver, spleen, lungs, and pancreas were extracted. The pancreas (pancreatic parenchyma plus tumor) was then weighed after being separated for the spleen. Organ samples were kept in formalin for 24 hours and then stored in 70% ethanol before paraffin inclusion.

## Results

**Figure 2:**
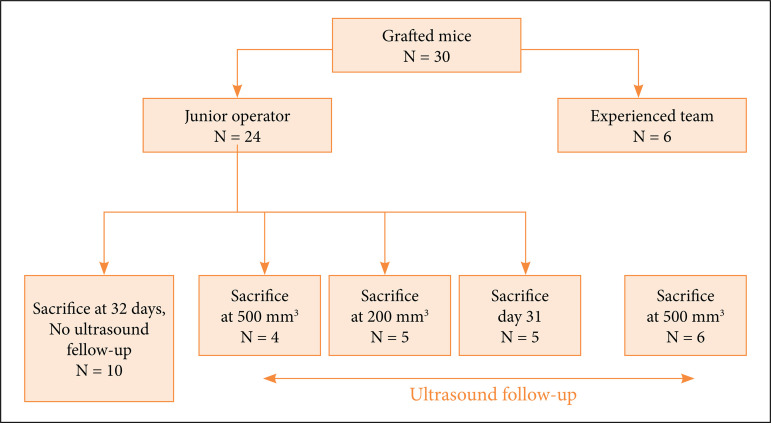
Schematic representation of the planned experiments of grafted mice and their follow-up.

Source: Authors.

Twenty-four mice were grafted by a junior (a master 2 student) and six by a senior operator (experienced member of the Centre de Recherche en Cancérologie de Toulouse team in Toulouse) (Fig. 2).

Over the 24 mice grafted by the junior operator, ten were sacrificed at POD 32 without ultrasound follow-up, four mice were sacrificed when the tumor reached 500 mm^3^, and 10 mice were sacrificed at intermediate endpoint–five were sacrificed when the tumor volume reached 200 mm^3^ and five at POD 31 independently of the tumor volume.

**Figure 3:**
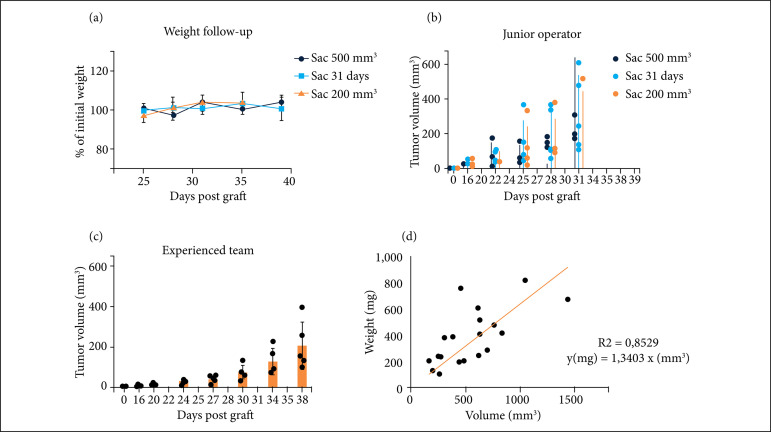
Mice follow up. **(a)** Mean weight and standard deviation based on initial weight (day 0): Sac 500 mm^3^ (blue): mice sacrificed when tumor volume reached 500 mm^3^, Sac 31 days (green): mice sacrificed at day 31, and Sac 200 mm^3^ (orange): mice sacrificed when tumor volume reached 200 mm^3^. **(b)** Mean tumoral volume and replicates estimated using ultrasound for each group, on the cohort grafted by a junior operator, Sac 500 mm^3^ (blue): mice sacrificed when tumor volume reached 500 mm^3^, Sac 31 days (green): mice sacrificed at day 31 and Sac 200 mm^3^ (orange): mice sacrificed when tumor volume reached 200 mm^3^. **(c)** Mean tumoral volume and replicates estimated using ultrasound on the cohort grafted by a senior team. **(d)** Correlation between tumor volume estimation using ultrasound and tumor weight and standard linear regression (y = 1,3403 x, R2 = 0,8529).

Source: Authors.

Over the twenty-four mice, twenty-tree (95,8%) mice developed a pancreatic tumor. No mice were sacrificed because of clinical worrisome feature or important weight lost. Weight was stable during the follow-up in each group (Fig. 3). Grafts in 11 mice (50%) were successful, nine mice (42%) presented a partial success and one (8%) failed. One mouse was sacrificed because of an elevated total tumor burden secondary to a large subcutaneous tumor (pancreatic tumor was 390 mm^3^ and subcutaneous tumor was 532 mm^3^). Eleven mice had subcutaneous tumors (42%). Over the 23 mice sacrificed after day 31, three (13%) had macroscopic liver metastasis. No macroscopic pulmonary or peritoneal carcinomatosis metastasis was found.

All subcutaneous tumors seen at sacrifice were localized at incision site and had been identified during ultrasound examination. Over the 14 mice for which tumor growth was followed by ultrasound, 85,7% of them presented a tumor at day 16 after grafting, and 100% at day 22. Mean tumor volume evolution for each group is represented in Fig. 2b, and each dot represents a mouse.

Regarding the six mice grafted by the senior operator, all of them developed a tumor, and two had a subcutaneous graft (33%), which resulted in 67% successful and 33% partially successful grafts.

Although there was some heterogeneity in tumor growth, both junior and experienced team tumor growths followed an exponential form (Figs. 2b and 2c). In both experimental procedures, the reported weight of the pancreas correlated with tumor volume measured at the day of sacrifice by ultrasound (Fig. 2d). Standard linear regression showed R^2^ = 0,8529.

## Discussion

Here we described our method for orthotopic pancreatic cancer graft and follow-up in a syngeneic and immunocompetent murine model. No previous experience in small animals’ surgery was required as shown by our results obtained from an inexperienced master student (50% totally successful and 42% partially successful grafts). Although most of the animals showed a subcutaneous tumor, only one was sacrificed because of this ectopic tumor burden. It was considered as a graft failure in our definition as it constituted a reason for early sacrifice. Our master student had a 95,8% of graft intake with the presence of subcutaneous tumors in 42% of the animals, while experienced operator reported 100% success with 30% subcutaneous tumors.

Mice’s weights were stable during the follow-up, showing good tolerance of the procedure. Interestingly, we report one mouse with an important weight lost which coincided with a tumor volume reaching almost 500 mm^3^ emphasizing the importance of this endpoint volume. Larger volume end points might have led to unnecessary animal discomfort.

In our study, the tumoral growth was following an exponential model which is consistent with previously reported studies^17^^-^ 19. However, depending on the cell line used (KPC6694c2 or Panc02) or the number of tumoral cells injected in the pancreas (varying from 5 × 10^4^ to 2,5 × 10^5^), the kinetics of the growth was slightly different compared to our model. Depending on the aggressiveness of the grafted cell line, on the suspected efficacity of the treatment tested and the aimed duration of the treatment, protocol should be adapted especially the number of injected cells. Authors may use a different cut-off based on the amount of material needed for further investigations or the time of treatment needed. The endpoint of the experiment could be based on a duration of the experiment, such as four^17^^,^ 19 or five weeks^18^ after grafting, or, as we did, could be based on a maximum tumor volume. Using tumor volume cut-off allowed a medium sacrifice at POD 39 (range 28-42).

Some authors rather use heterotopic grafting (subcutaneous) as tumoral growth can be directly followed up by palpation and measured with a caliper^13^. However, using orthotopic growth with a short training on ultrasound seems to be a robust model. Indeed, we showed good correlation between the tumor weight at sacrifice and the tumor volume calculated with ultrasound. This is important as it validates the efficiency and relevance of the ultrasound technic used for the mice follow-up.

Moreover, while heterotopic graft is methodically easier, it was demonstrated to be less robust for studying drug efficacy. Treatment efficiency differed due to the difference in vascularization system^13^^,^ 20. Besides, heterotopic grafting does not confront the tumoral cells with their usual surrounding microenvironment (cancer associated fibroblasts, extracellular matrices, and immune cells essentially). It is now well established that the microenvironment strongly interacts with the tumor^15^ to support and promote tumor growth, through epithelial-to-mesenchymal transition^21^ or metastasis development^16^. Also, local and specific microbiome will differ between the two grafting methods, and recent studies have shown that local microbiome with lower diversity is associated with worse outcome^22^.

Different models for therapeutic screening are available; every model has its own advantages and limitations. Combination of these models is useful: for example, spheroid and organoid models are used for drug testing as they can reduce the number of animals needed necessary for drug validation^23^. Mouse models remain a key model in the step-up approach for drug validation. Herein we described a reproductible method for orthotopic and syngeneic pancreatic cancer cell in immunocompetent mice. Extrapolation of this technique with human tumoral cells could be carried out in nude mice.

## Conclusion

Therapeutic innovations are needed to improve pancreatic cancer treatment. Murine models remain a good tool to recreate, in pre-clinical studies, the complexity of pancreatic cancer environment. In this study, we described a robust and reproducible method for grafting pancreatic cancer cells in an immunocompetent mouse model. We also described a useful method of ultrasound for an efficient tumor growth evaluation. This method is reliable and can be performed by a junior student after a short training.

## Data Availability

The authors confirm that the data supporting the findings of this study are available within the article [and/or] its supplementary materials.
